# Evidence for a heritable predisposition to Chronic Fatigue Syndrome

**DOI:** 10.1186/1471-2377-11-62

**Published:** 2011-05-27

**Authors:** Frederick Albright, Kathleen Light, Alan Light, Lucinda Bateman, Lisa A Cannon-Albright

**Affiliations:** 1 Pharmacotherapy Outcomes Research Center, Department of Pharmacotherapy, College of Pharmacy, University of Utah, USA; 2 Department of Anaesthesiology, University of Utah, USA; 3 Fatigue Consultation Clinic, Salt Lake City, UT, USA; 4 Division of Genetic Epidemiology, Department of Internal Medicine, School of Medicine, University of Utah, USA; 5 George E. Wahlen Department of Veterans Affairs Medical Center, Salt Lake City, Utah, USA

## Abstract

**Background:**

Chronic Fatigue Syndrome (CFS) came to attention in the 1980s, but initial investigations did not find organic causes. Now decades later, the etiology of CFS has yet to be understood, and the role of genetic predisposition in CFS remains controversial. Recent reports of CFS association with the retrovirus xenotropic murine leukemic virus-related virus (XMRV) or other murine leukemia related retroviruses (MLV) might also suggest underlying genetic implications within the host immune system.

**Methods:**

We present analyses of familial clustering of CFS in a computerized genealogical resource linking multiple generations of genealogy data with medical diagnosis data of a large Utah health care system. We compare pair-wise relatedness among cases to expected relatedness in the Utah population, and we estimate risk for CFS for first, second, and third degree relatives of CFS cases.

**Results:**

We observed significant excess relatedness of CFS cases compared to that expected in this population. Significant excess relatedness was observed for both close (p <0.001) and distant relationships (p = 0.010). We also observed significant excess CFS relative risk among first (2.70, 95% CI: 1.56-4.66), second (2.34, 95% CI: 1.31-4.19), and third degree relatives (1.93, 95% CI: 1.21-3.07).

**Conclusions:**

These analyses provide strong support for a heritable contribution to predisposition to Chronic Fatigue Syndrome. A population of high-risk CFS pedigrees has been identified, the study of which may provide additional understanding.

## Background

The Chronic Fatigue Syndrome (CFS) disease classification attempts to identify patients with a condition distinct from the fatigue of other chronic illness. It is most frequently defined by the Fukuda definition: severe, unexplained, chronic fatigue lasting at least 6 months, accompanied by 4 of 8 symptom criteria (post-exertional malaise, impaired memory or concentration, unrefreshing sleep, muscle pain, arthralgia, headaches, sore throat or, tender cervical or axillary lymph nodes)[1,2]. According to population based studies, 800,000 or more people in the US meet these criteria [3-8] or have CFS like disease [8]. The Center for Disease Control (CDC) posts that the illness costs the US $9 billion annually in lost earnings and productivity [9,10].

CFS is a complex illness that is not well understood. This applies to the genetic and environmental origins of CFS, as well as factors that may contribute to the severity of symptoms and outcomes. Despite efforts to develop standardized research criteria to define the disease, progress in CFS diagnosis and treatment has been slow, in part due to concurrence in origins, causation, lack of a standard clinical definition, and lack of specific biomarkers. A community-based screening study by the CDC indicated that only 9%-16% of individuals with CFS had been diagnosed. Variations in presentation (age of onset, sudden versus gradual onset, and co-morbid conditions) further increase the heterogeneity of CFS patients. It has been proposed that predisposition to CFS may have a genetic basis, although environmental factors, viral illnesses, stressful life events or traumas may also be implicated [4,6,8,11,12]. CFS has been described as a disorder of the brain, the immune system, or the endocrine system; studies have documented underlying pathophysiological processes involving all of these systems [13-18].

There are numerous studies suggesting that genes might play a role in the development of CFS or in its progression [19-21]. Several twin studies have examined genetic vs. environmental determinants of CFS [21-24]. These studies have shown higher concordance in monozygotic vs. dizygotic twins. A small familial aggregation study (CFS cases = 25), showed excess relative risks for CFS in first-degree relatives of CFS cases compared to controls [20].

Analysis of a heritable contribution to CFS is confounded by the newness of the diagnosis, the lack of specific diagnostic criteria or biomarkers, and high rates of co-morbidity with other syndromes. Here we report the existence of a unique, population-based resource with genealogical information linked to diagnosis data allowing analysis of the genetic relationships among 811 individuals diagnosed with CFS since 1993. The combined data have been analyzed to examine the hypothesis of a heritable contribution to CFS.

## Methods

The original Utah Population Database (UPDB) represented genealogical records of Utah pioneers and their descendants. The UPDB was created from family group sheets compiled by members of the Church of Jesus Christ of Latter-day Saints (LDS) submitted to the Genealogical Society of Utah. Researchers at the University of Utah computerized these genealogical paper records of Mormon pioneers and their descendants into an electronic database [25].

The original UPDB genealogy represented approximately1.6 million individuals in genealogies up to 6 generations deep. Genealogical data added since the original derives from state records (birth, death, driver's license and others) entailing 6.5 million individuals. New individuals from these sources who link to the original genealogy data produce pedigrees up to 15 generations deep with 2.3 million individuals belonging to genealogies at least 3 generations deep.

Numerous Utah data sets have been linked to UPDB records, including Utah Cancer Registry (UCR) records. The UPDB has also been linked to individual medical data from the University of Utah Health Sciences Hospital (UUHSH) Enterprise Data Resource Center. The UUHSH Data Warehouse (DW) contains comprehensive hospital, medical, and clinical data from 1993 to present. The UUHSH serves 20% of the state's residents. The UUHSH Data Resource Center currently contains over 1.8 million patient demographic records for patients treated at the hospital and outpatient clinics. Over 1.26 million (73%) of these hospital records have been matched to a "person record" in UPDB.

CFS patients were identified by the presence in the medical record of a CFS-specific ICD-9 diagnostic code (780.71) originating from medical providers involved in the patient's care. Although in other areas of the country the rate of CFS diagnosis may be lower, it is likely that in Utah the appropriate application of the Fukuda definition and diagnosis of CFS increases the rate. This is likely related to the CFS referral clinic in the community, and the availability of CFS-specific Continuing Medical Education to community providers during the last decade. UUHSH has no clinics specifically devoted to CFS, or specialists who claim expertise in CFS.

Although all University of Utah hospital and clinic records have been linked to the UPDB genealogy, only a selected subset of these records can be accessed, due to the sensitive nature of the data. A set of randomly selected hospital patients was identified for use as controls, representing 20% of all University of Utah hospital/clinic patients who also had linked genealogy data (n~200,000). We created cohorts to which these UUHSC patients were assigned, and from which controls were selected randomly. All individuals were assigned to 1 of 132 cohorts based on sex, birth-year, and birthplace (i.e., Utah or not). All controls for the analyses performed were selected randomly from this set.

This unique resource allowed us to identify all genetic relationships between patients with a diagnosis of CFS. To examine evidence for a genetic contribution to CFS in the UPDB, two different analyses were performed: estimation of relative risks in close and distant relatives of CFS cases, and comparison of average relatedness in CFS cases to controls.

### Relative Risks in Relatives

We estimated relative risk (RR) for CFS among the relatives of patients using the classical odds ratio (OR) method. Although we were able to identify all patients with a diagnosis of CFS, we were only able to identify 20% of all known UUHSC patients in any given cohort, resulting in an inability to estimate true CFS population rates. We therefore selected 5 matched (hospital) controls for each case for RR estimation. Because of the over-representation of cases in each cohort, we excluded cases from selection as controls.

To estimate the RR we compared the number of CFS cases among the relatives of cases to the number of CFS cases among the relatives of 5 independently selected sets of matched UUHSC controls (relatives counted without duplication). Because of the nature of the UPDB resource, and the fact that we identify all genetic relationships between all CFS cases, this study did not require any screening of relatives; we identified all relatives of all cases (and all relatives of matched controls) using genealogy data, and then counted UUHSC CFS cases who were identified among these relatives. The significance of the test of the null hypothesis RR = 1.0 was determined by Fisher's Exact Test for the 2 × 2 table. Confidence intervals for the RR were estimated as described in Agresti [26]. This method has been applied to other disease phenotypes in the UPDB [27,28].

### Genealogical Index of Familiality (GIF)

We used the Genealogical Index of Familiality (GIF) statistic to test the hypothesis of no excess relatedness among CFS cases. This statistic, developed for use with the UPDB [29,30], measures the average pair-wise relatedness of a set of individuals, and compares the measure to the average expected relatedness of a set of similar individuals in this population. In contrast to the RR, which examines close relationships between cases, the GIF analysis considers all pair-wise genetic relationships between all cases, and separately for matched controls.

The GIF relatedness measure for a pair of individuals implements the Malécot coefficient of kinship [31], which is defined as the probability that randomly selected homologous genes from the 2 individuals are identical by descent from a common ancestor. For parent/child the coefficient is 0.50 (1/2), for siblings or grandparents the coefficient is 0.25 (1/4), for avunculars the coefficient is 0.125 (1/8), and so forth. The contribution to the GIF statistic is smaller for pairs with a greater genetic distance between them.

The case-GIF statistic is defined as the average of the coefficients of kinship between all possible pairs of CFS cases (x 100,000). A control-GIF statistic is calculated as the average of the coefficients of kinship between all possible pairs in a single set of randomly selected matched controls. Comparison of the average relatedness of a set of cases to the distribution of the average relatedness statistics estimated for 1,000 independent sets of matched controls, provides an empirical test of significance.

Because both close and distant relationships are observed, the GIF statistic will show excess relatedness in the presence of genetic effects only, or environmental (familial, but non-genetic) effects only, or in the presence of a combination of both effects. The overall GIF statistic therefore tests the alternative hypothesis of no excess familial clustering of any origin. We also utilized the GIF test to examine the more specific hypothesis of a genetic contribution. To do this, the GIF statistic was estimated while ignoring all close relationships (relationships with genetic distance < 4, or closer than first cousins). We term this statistic the distant GIF (dGIF). The dGIF statistic allows a test of the hypothesis that there are significantly more distant pair-wise relationships observed among cases than would be expected, providing strong support for a familial effect that is based on shared genes.

No patient identifiers were used in this study; analysis of genetic relationships between affected individuals is non-identifiable. The University of Utah Institutional Review Board and the Utah Resource for Genetic and Epidemiological Research (RGE) approved the utilization of UPDB data in this study.

## Results and Discussion

We identified 941 patients with a CFS diagnosis in the UUHSC DW from 1993 to present that also had Utah genealogy data. Age at diagnosis was estimated using the minimum admission date for a CFS diagnosis, and thus may be over estimated. Twelve percent of the cases had an age at diagnosis less than age 20 years; 28% of the cases had an age at diagnosis greater than 59 years; 653 (69%) were female. Because CFS is associated with various co-morbid conditions, with possible genetic associations, we excluded the 130 CFS cases that also had a diagnosis of cancer in the UCR. This left 811 CFS cases for analysis.

### Relative Risks

Table 1 summarizes the RR estimates risk of CFS among first, second, and third degree relatives of CFS cases. For each degree of relationship considered, Table 1 shows the number of relatives considered (for cases and controls), the number of CFS cases observed among the relatives (for cases and controls), the significance for the Fisher's Exact test, the Odds Ratio (OR) estimate of RR, and the 95% confidence interval for the RR. Relative risks for first (RR = 2.70), second (RR = 2.34), and third degree (RR = 1.93) relatives were significantly elevated.

**Table 1:** Estimated RRs for first-, second-, and third-degree relatives in the 811 CFS cases

Degree relative	Relatives of: cases / controls	# CFS cases in relatives of: Cases / controls	p-value	RR	95% CI
first	5,573 / 28,965	19 / 37	0.001	2.70	1.56, 4.66
second	15,469 / 80,206	16 / 36	0.008	2.34	1.32, 4.19
third	39,766 / 201,717	24 / 64	0.009	1.93	1.21, 3.07

### Genealogical Index of Familiality

**Figure 1:**
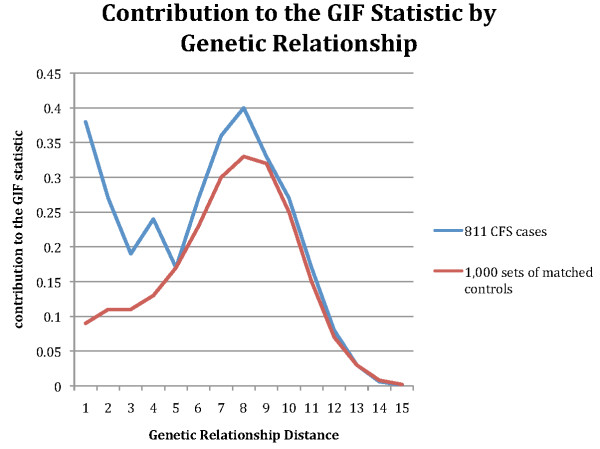
**Contribution to the GIF statistic, measuring average relatedness**. Contribution to the GIF statistic (y-axis) by genetic relationship (genetic distance, x axis) between all pairs of related CFS cases compared to all pairs of related controls. Genetic distance 1 = parent/offspring, 2-siblings or grandparent/grandchild, 3 = avunculars, and so forth).

Table 2 shows the results of the GIF test for excess relatedness in the 811 CFS cases, including the average relatedness of the cases, the mean average relatedness for 1000 sets of matched controls, the empirical significance of the overall test for excess relatedness, and the empirical significance of the dGIF. The average relatedness of CFS cases was significantly greater than expected when all relationships were considered (p <0.001), showing strong evidence for excess familial clustering (due to either shared environment, or shared genes, or a combination). When close relationships (genetic distance <4) were ignored, the significant excess was still present (p = 0.010). Figure 1 displays the GIF test graphically. It shows the contribution to the GIF statistic (y-axis) by the relationship (x-axis; genetic distance) between all pairs for CFS cases (and controls). This observation of significant excess relationships observed at almost all degrees of relationship strongly supports a genetic contribution to predisposition to CFS.

**Table 2:** GIF test for excess relatedness among 811 CFS cases

Phenotype	n	Case Average Relatedness	Mean Control Average Relatedness	Empirical p-value GIF	Empirical p-value Distant GIF
CFS	811	3.15	2.31	< 0.001	0.010

## Conclusions

The existence of a Utah resource combining up to 15 generations of genealogy data with medical diagnosis data from 1993 has allowed testing of the hypothesis of a heritable contribution to CFS. The methods used in this study have previously provided evidence for a heritable component to many diseases, including: prostate cancer, influenza mortality, aneurysm, cancer, rotator cuff disease, asthma mortality, and diabetes, among others [28,32-34]. Studies of Utah high-risk pedigrees identified in the UPDB have lead to the discovery of multiple cancer predisposition genes including *BRCA1, BRCA2, p16*, and *HPC2/ELAC2 *[35-38]. The UPDB data analyzed represents a homogeneous population that has been shown to be genetically representative of Northern Europe, with normal U.S. inbreeding levels [39,40].

Significantly increased risks among first degree relatives are often referred to as providing evidence for a "genetic" contribution to disease. However, given the sharing among close relatives of their genes, lifestyle, and environment, increased first degree risk may simply indicate familial clustering, it does not provide evidence for a genetic contribution. However, significant excess risks in second and third degree relatives strongly indicates a genetic contribution to disease, given the much lower likelihood of these relatives sharing common risks and environments.

Analysis of CFS in a large Utah resource shows clear evidence of significant excess familial clustering and significantly elevated risks for CFS among first, second, and third degree relatives of CFS cases. The results strongly support a genetic contribution to predisposition to CFS as it is currently defined and diagnosed by clinicians in Utah. Although a genetic predisposition to CFS has been suggested in the literature, this is the first population-based analysis to comprehensively support this claim.

This study used a uniform, consistent source for all diagnoses, and is not limited by bias introduced by study designs involving selected ascertainment of cases or requiring recall for diagnoses. The most significant limitation of this analysis is the narrow window of view to identify individuals diagnosed with CFS. This results from the relatively short period of time for which this diagnosis has existed, and the limited time-period of diagnosis data available (1993-present). These effects limit our ability to identify cases who might be related across different generations (e.g. grandparent/grandchild or avunculars). Although CFS cases may have been censored from our observation in this resource, cases are uniformly censored across the resource, leading to conservative, but unbiased, estimates of familiality.

We excluded CFS cases with a cancer diagnosis, which might have been a cause of CFS symptoms in these cases. Other potential confounders could not be considered: including other heritable predisposing conditions (e.g., depression), or risk factors that are familial, but not genetic (e.g., occupation, socioeconomic factors, or healthcare access).

This study of CFS heritability does not allow determination of the mechanisms that lead to predisposition to CFS. We have identified multiple pedigrees with a significant excess of CFS cases. We propose study of these pedigrees to identify the gene(s) predisposing to CFS, as well as to better understand mechanisms and potential environmental factors and triggers.

Studies reporting an association of CFS with XMRV and MLV-related viruses have provided conflicting results [41-44]. While association of CFS with an infectious-like syndrome at onset is recognized, and many microbial and viral infections have been implicated as possible triggers, no single agent has been associated with a large fraction of cases. It might be hypothesized that a heritable predisposition to virus infection explains both our findings and the complex virus associations that have been recognized.

Identification of CFS predisposition genes, and increased understanding of how these genes affect health could allow identification of predisposed individuals at an earlier age, prophylactic screening for at-risk individuals, improved healthcare standards to reduce risk of CFS development, all leading to identification of treatments or medications that could prevent or delay onset of symptoms in those impaired by this debilitating disease.

## Competing interests

No authors have any financial involvement or affiliation with any organization whose financial interests may be affected by material in the manuscript or which might potentially bias it.

## Authors' contributions

Each author contributed equally to the development and ensuing research of the project. Each author made significant and substantial written contributions in this manuscript. All authors read and approved the final version of the manuscript.

## Pre-publication history

The pre-publication history for this paper can be accessed here:

http://www.biomedcentral.com/1471-2377/11/62/prepub
